# Efficacy assessment of methylcellulose-based thermoresponsive hydrogels loaded with gallium acetylacetonate in osteoclastic bone resorption

**DOI:** 10.1007/s13346-023-01336-5

**Published:** 2023-04-04

**Authors:** Pratyusha Ghanta, Timothy Winschel, Evin Hessel, Oluyinka Oyewumi, Tori Czech, Moses O. Oyewumi

**Affiliations:** 1 Advanced Drug Delivery Laboratory, Department of Pharmaceutical Sciences, College of Pharmacy, Northeast Ohio Medical University, 4209 State Route 44, Rootstown, OH 44272 USA; 2 Department of Biomedical Sciences, Kent State University, Kent, OH 44240 USA; 3 Department of Geological Sciences, Central Connecticut State University, New Britain, CT 06050 USA

**Keywords:** Osteoclasts, Osteoblasts, Gallium, Drug delivery, Osteoporosis, Fractures

## Abstract

**Abstract:**

Homeostatic imbalance involving progressive stimulation of osteoclast (OC) differentiation and function will lead to an increased risk of fragility fractures. In this regard, we investigated gallium acetylacetonate (GaAcAc) as a possible treatment for osteoclastic bone resorption. Further, the extent to which suitable delivery systems can enhance the therapeutic potential of GaAcAc was evaluated. GaAcAc solution (10–50 µg/mL) suppressed OC differentiation using murine monocytic RAW 264.7 or hematopoietic stem cells. Methylcellulose-based hydrogels were fabricated and characterized based on biocompatibility with bone cells, GaAcAc loading, and thermoresponsive behavior using storage (G′) and loss (G″) moduli parameters. Compared to GaAcAc solution, hydrogels loaded with GaAcAc (GaMH) were more effective in suppressing OC differentiation and function. The number and extent of bone resorption pits from ex vivo studies were markedly reduced with GaMH treatment. Mechanistic assessment of GaMH efficacy showed superiority, compared to GaAcAc solution, in downregulating the expression of key markers involved in mediating OC differentiation (such as NFAT2, cFos, TRAF6, and TRAP) as well as in bone resorption by OCs (cathepsin K or CTSK). Additional studies (in vitro and in vivo) suggested that the performance of GaMH could be ascribed to controlled release of GaAcAc and the ability to achieve prolonged bio-retention after injection in BALB/c mice, which plausibly maximized the therapeutic impact of GaAcAc. Overall, the work demonstrated, for the first time, the therapeutic efficacy of GaAcAc and the therapeutic potential of GaMH delivery systems in osteoclastic bone resorption.

**Graphical Abstract:**

**Supplementary Information:**

The online version contains supplementary material available at 10.1007/s13346-023-01336-5.

## Introduction

Bone is a dynamic and mineralized connective tissue that functions through a cyclic homeostatic remodeling and resorption process, sustained throughout life [1, 2]. Osteoblasts (OBs) are a category of bone cells responsible for secreting organic matrix and depositing minerals to create new bone. In contrast, osteoclasts (OCs), multinucleated bone cells derived from hematopoietic precursors, are directly involved in the resorption of the bone matrix [3]. The functioning of these bone cells is convoluted where OBs secrete cytokines like macrophage colony–stimulating factor (M-CSF) and receptor activator of NFκB ligand (RANKL) that stimulate OC differentiation and function during the active remodeling phase of bone regeneration [4–6]. Essentially, potential disruption in either OBs or OCs will lead to an imbalance in bone homeostasis. Such homeostatic imbalance can lead to skeletal fragility or abnormal bone formation depending on whether OC or OB functions are enhanced or suppressed [2].

Specifically, OCs are differentiated multinucleated cells from mononuclear cells of the hematopoietic stem cell lineage. The differentiation process occurs in the presence of many factors, mainly macrophage colony–stimulating factors (M-CSF), secreted by osteoprogenitor mesenchymal cells; and receptor activation of nuclear factor kappa-B ligand (RANKL), which is secreted by OBs, osteocytes (OCTs), and stroma cells [1, 2, 5]. Osteoclastogenesis (production of OCs) has been reported to be regulated through the pathways involving NF-κB, cFos, JNK, etc. [7]. Upon complementary binding of RANKL to RANK receptor, TRAF6 is activated via phosphorylation, and in turn, transcription factors such as cFos and NFAT2 are stimulated [7]. cFos is present in the initial stages of the OC differentiation and later regulates the auto-amplification of NFAT2 and other OC markers [8]. Mature OCs bind tightly to the bone and create a sealed microenvironment where they produce collagenolytic enzymes like cathepsin k (CTSK) and other secreted protons that affect the organic matrix and degrade the mineral component [9, 10]. Interestingly, OBs, OCT, and stromal cells secrete factors to stimulate osteoclastogenesis; however, osteoprotegerin (OPG), produced by the same cells, inhibits osteoclastogenesis through negative feedback [1, 11, 12].

In most bone resorptive diseases, the attributable mechanisms could result from either downregulation of OPG or upregulation of RANKL and M-CSF or other factors that contribute to the imbalance in the bone homeostasis [13]. Potential disruption of the function of OC or OB will interfere with the process of bone remodeling, which is crucial in conditions such as fractures, skeletal adaptation, and calcium homeostasis [1]. An imbalance in this process can lead to severe bone-related disorders. For instance, excessive resorption by overstimulated OCs can contribute to excessive bone loss, resulting in disorders such as osteoporosis and Paget’s disease [14]. The impact of overstimulated osteoclastic bone resorption is associated with skeletal fragility and a high propensity for fracture [15], notable features of osteoporosis [16, 17].

Osteoporosis contributes heavily to the 1.5 million fractures that occur yearly in the USA [18]. Therapeutic agents that inhibit relevant pathways/targets associated with osteoclastic bone resorption are highly sought after in the management of osteoporosis or similar diseases. It is desirable to prevent fracture through impeding bone resorption cascade which could be combined with facilitation of bone regeneration [19]. Examples of therapeutic agents that can aid in bone regeneration include teriparatide (Forteo®), and hormone replacement therapy with estrogen, calcitonin, and bisphosphonates [20, 21]. However, there are limitations surrounding the routine use of these products, such as high cost, poor compliance, risks, and side effects, mixed results, and poor pharmacokinetic profile without other modulating agents [22]. The effectiveness of other approved osteinductive agents such as bone morphogenic proteins 2 and 7 has been plagued by extensive adverse events such as post-operative inflammation and adipogenesis [23, 24]. There is a need for effective therapeutic agents to manage osteoclastic bone resorption while maintaining attractive safety profiles.

Meanwhile, the anti-resorptive activity of gallium nitrate has gained much attention with the approval of its clinical use in treating malignancy-associated hypercalcemia [25, 26]. Further, gallium nitrate has been shown to inhibit osteoclastogenesis, without detectable adverse effects on bone cells [27, 28]. Other studies have investigated application of gallium nitrate as a component of calcium phosphate biomaterials for bone grafting or reconstructive surgery [29, 30]. Mechanistic studies revealed that gallium nitrate can inhibit various signaling pathways that are linked to OC differentiation and bone resorption such as tartrate-resistant acid phosphatase (TRAP), cathepsin K (CTSK), RANK, OC-Stamp, NFAT2, NFκB, calcitonin receptor (CTR), and transient receptor potential cation channel subfamily V (TRPV5) [27, 28, 31].

We believe that the gallium compound holds great potential as a safe and effective treatment option for osteoclastic bone resorption and is worthy of further investigation. Meanwhile, the potential application of gallium compounds will require suitable delivery systems that enhance retention at the bone site and decrease off-target distribution while enhancing efficacy. We are unaware of previous work on delivery systems for applying gallium compounds in osteoclastic bone resorption. Thus, this study aims to investigate the efficacy of gallium acetylacetonate (GaAcAc) as a new gallium compound for potential application in osteoclastic bone resorption. We also propose to fabricate and assess the effectiveness of suitable delivery systems using in vitro and in vivo approaches.

We specifically set out to assess thermoresponsive hydrogels as delivery systems for GaAcAc. Unique characteristics of hydrogels can be attributed to the type of materials used in preparation and method of synthesis as well as the ability to respond to changes in the environment like temperature, pH, or the presence of enzymes and specific biomarkers [32]. We pay particular attention to thermoresponsive hydrogels that respond to changes in temperature by converting from solution at room temperature to gel at body temperature [33]. The attractiveness of thermoresponsive hydrogels is that they can be easily injected locally into the desired bone site, at room temperature in the form of a solution and transition to gel at the bone site with temperature change, thereby localizing the therapeutic agent at the desired bone site [34, 35].

The biocompatibility and affordability of cellulose-based hydrogels make them a great choice for our study [36]. In general, cellulose-based hydrogels are formed by crosslinking aqueous solutions of cellulose ethers such as methylcellulose and hydroxypropyl methylcellulose [37]. Cellulose-derived hydrogels form a mesh-like network that traps the drug moieties within its structure until the surrounding environment enables its release in a controlled manner [38, 39].

Thus, we fabricated methylcellulose-based hydrogels that were evaluated as delivery systems for GaAcAc based on (i) biocompatibility towards pre-OB and pre-OC cells, (ii) effects of GaAcAc loading on the hydrogel’s thermoresponsiveness, and (iii) efficacy assessment in inhibiting OC differentiation and function. Overall, we applied in vitro and in vivo approaches to assess the suitability of methylcellulose-based hydrogels as a delivery for GaAcAc in osteoclastic bone resorption.

## Experimental

### Materials

RAW 264.7 (murine macrophage-osteoclast precursor) cells were procured from ATCC, Manassas, VA. Gallium (III) acetylacetonate (GaAcAc, MW 367.05 g/mol) was procured from Strem Chemicals (Newburyport, MA), methylcellulose powder, penicillin–streptomycin, 3-(4,5-dimethylthiazol-2yl)-2,5-diphenyltetrazolium bromide (MTT), Fast Red Violet Salt, toluidine blue and naphthol AX-MX, protease inhibitor cocktail, neutral red dye, and 2-mercaptoethanol were all obtained from Sigma-Aldrich (St. Louis, MO). Dimethyl sulfoxide (DMSO), phosphate-buffered saline (PBS), bovine serum albumin (protease free), alpha-modified eagle’s medium (α-MEM), Dulbecco’s Modified Eagle’s Medium (DMEM), and *p*-nitrophenyl phosphate were all purchased from GIBCO & Fisher Scientific (Pittsburgh, PA). RANKL and M-CSF were bought from R&D Technologies (Minneapolis, MN), and fetal bovine serum (FBS) was obtained from Atlanta Biologicals (Lawrenceville, GA). Bovine cortical bone slices were purchased from BioVendor R&D® (Asheville, NC). NFAT2, TRAF6, cFos and IgG-HRP, and GAPDH antibodies were procured from Cell Signaling Technology (Danvers, MA), and TRAP antibody from Abcam (Cambridge, UK). Indocyanine green (ICG) was purchased from Cayman Chemical Company (Ann Arbor, MI). Gallium (III) nitrate hydrate (MW 255.74 g/mol) and RIPA lysis/extraction buffer were purchased from ThermoFisher Scientific (Waltham, MA), 24-well transwell plates were procured from Corning Inc. (Corning, NY), Immobilon ® Forte Western HRP substrate was procured from Millipore (Burlington, MA). 4 × laemmli buffer, TransBlot ® Turbo ™ Mini-size PVDF membrane, Trans-Blot Turbo Mini-size Transfer Stacks, and 10 × Transfer buffer were procured from Bio-Rad (Hercules, CA). Immunoblotto non-fat dry milk was procured from Santa Cruz Biotechnology (Dallas, TX), and Immobilon® Forte Western HRP substrate was procured from EMD Millipore (Burlington, MA). BCA Pierce Assay kit was procured from Thermo Scientific Pierce™, Rockford, IL. Nitric acid (65% v/v) was procured from Fluka Analytics (Buchs, CH), and Yttrium and ICP grade water were procured from Inorganic Ventures (Christiansburg, VA). Mouse CTSK/Cathepsin K (Sandwich ELISA) ELISA Kit was procured from Lifespan Biosciences (Seattle, WA).

### Preparation of methylcellulose hydrogel (MH) and GaAcAc-loaded hydrogels (GaMH)

Methylcellulose hydrogels (MH) were prepared via the dispersion technique by adapting earlier reported methods [40, 41]. Briefly, the methylcellulose powder was weighed and dispersed in preheated (55 °C) PBS (2–12% w/v), followed by overnight spinning (700 RPM) at 4 °C. The resultant gels were maintained in 4 °C before further testing. The same procedure was followed to prepare GaAcAc-loaded hydrogels (GaMH) with a slight modification of dropwise addition of various concentrations of GaAcAc into the hydrogels under stirring.

### Rheological measurements

Rheological characterization of different concentrations of MH and GaMH with different concentrations of GaAcAc was analyzed using a Haake Mars Rheometer (222–1912, Thermo Fisher Scientific). The following parameters were conducted: dynamic shear oscillation and temperature-dependent analysis of modules. The measurements were carried out using minimal strain to characterize the viscoelastic properties using serrated plates of 40 mm diameter with a plate-to-plate distance of 0.9 mm. Temperature-dependent studies of the storage (G′) and loss (G″) modulus were conducted using oscillatory shear with temperatures ranging from 20 to 50 °C (heating rate: 2 °C/ min) at a constant frequency (1 Hz) and shear strain (1 mrad). G′ is the measure of elasticity of the material and its ability to store energy, whereas G″ is the ability is to release energy. By measuring the G′ and G″ moduli over a temperature range, the sol–gel transition of the hydrogel can be assessed [42]. The point at which G′ is equal to G″ is defined as gelation temperature, indicating the hydrogel’s phase transition due to its response to the change in temperature [40]. The data was analyzed and presented with the help of Rheology Data Manager software. Based on the data obtained, the percentage of methylcellulose hydrogel and drug-loaded hydrogel were selected for further analysis [43].

### Gelation test of hydrogel

The test-tube tilting method was applied to observe the ability of the hydrogels to undergo reversible solution-to-gel transition at different temperature conditions. Starting with 4 °C, the gel was sealed in a glass vial. The phase of the gel was observed by tilting the vial several times. The process was repeated at 25 °C and 37 °C [36, 43].

### In vitro degradation of MH hydrogel

To characterize the in vitro degradation of MH, we maintained the hydrogels at 37 °C and measured the initial weight of the solidified gel. Subsequently, we added 1 mL of PBS (pre-warmed at 37 °C) on top of the solidified gel and maintained it at 37 °C over time. At pre-determined time intervals, we aspirated the PBS layer on top of the solidified gel before measuring the weight of the hydrogel layer over time. The extent of MH hydrogel degradation was calculated using the initial weight of the hydrogel layer and the weight measured at different time intervals during the experiment [36, 43].

### In vitro GaAcAc release from hydrogel

The rate and extent of GaAcAc release from GaMH were assessed. Briefly, we maintained GaMH at 37 °C. Subsequently, we added 1 mL of pre-warmed (37 °C) PBS on top of the solidified gel and subjected it to gentle agitation of 50 rpm in an incubator maintained at 37 °C. The PBS layer was aspirated at different time intervals and replenished with fresh pre-warmed PBS. The amount of GaAcAc released at various time points was analyzed by UV absorbance 278 nm.

### Cell culture

RAW 264.7 (pre-osteoclast) and MC3T3 (pre-osteoblast) cells were cultured and maintained in DMEM and AMEM supplemented with 10% FBS and 1% PenStrep in a humidified incubator at 37 °C and 5% CO_2_ conditions. RAW and MC3T3 cells, upon 70–80% confluency, were gently detached using a cell scraper and/or 0.25% v/v trypsin/EDTA. The detached cell suspension was collected and centrifuged at 1500 RPM for 5 min and seeded accordingly to the requirement for each experiment.

### Biocompatibility assessment

We assessed the biocompatibility of GaAcAc in pre-osteoclastic cells (RAW 264.7) and pre-OB cells (MC3T3) in pre-OC cells (RAW 264.7). Cells were incubated (37 °C) with various concentrations of GaAcAc (10–50 µg/mL; the corresponding molar concentrations are shown in Supplemental Table S1). After the stipulated incubation time, cell viability was measured by MTT assay using untreated control cells as a reference.

The biocompatibility of MH was carried out in pre-OC cells (RAW 264.7) by either incubating MH leachates with cells or introducing MH to cells via transwell inserts. To collect MH leachates, 1 mL serum-free media was added to solid hydrogels and incubated (37 °C) for 24 h. The supernatant was collected and filtered. RAW 264.7 cells were plated and incubated overnight in a complete growth medium. Subsequently, the content of each well was aspirated and treated with MH-filtered leachate supplemented with FBS and incubated for a pre-determined time. The percentage of cell viability was assessed using untreated control cells as a reference.

To add MH to cells via transwell inserts, cells were seeded overnight at a density of 5000 cells/well and incubated overnight. The next day, 100 µL of MH was added to the top compartment of the set and incubated for gelation before assembling the top and bottom compartments of the transwell plate. The assembly was then incubated for 24, 48, and 72 h. After each time point, the contents of each well were aspirated, and the cell viability assessment was carried out via MTT. The percentage of cell viability was calculated based on control (untreated) cells as a reference (100% viability).

### OC differentiation studies

#### RAW 264.7 cells differentiated OCs

RAW 264.7 (pre-OC) cells were seeded at a density of 1.5 × 10^5^ cells/cm^2^ (day 0) in a 24-well bottom dish of an insert plate and incubated overnight for adherence [44]. Subsequently (day 1), cells were treated with growth media supplemented with 30 ng/mL of RANKL (positive control) alone or RANKL (30 ng/mL) together with various treatments—GaAcAc solution, MH, or GaMH. Cells that did not receive RANKL supplementation served as negative controls. After the initial treatment and RANKL supplementation, cells in all treatment groups (except untreated control) received RANKL supplementation on day 3 to induce OC formation. On day 4, mature OCs were fixed at 4% paraformaldehyde for 10 min at room temperature and evaluated for the extent of OC differentiation based on tartrate-resistant acid phosphatase (TRAP) activity and stained with TRAP staining solution. TRAP (pink colored) stained and multinucleated (≥ 3 nuclei) cells according to earlier reported methods [45].

#### Hematopoietic stem cells differentiated OCs

All mice were housed and maintained at Northeast Ohio Medical University according to the Institutional Animal Care and Use Committee guidelines. Bone marrow cells were flushed from the femora, and tibiae were extracted from euthanized C57BL/6 mice (male, 6–8 weeks) as described previously. The flushed cells were incubated overnight to separate hematopoietic (in supernatant) and mesenchymal (adherent) stem cells. The supernatant was then collected and seeded at the required density based on the type of well plate used (96-well plate: 2 × 10^5^ cells/well and 24-well plate: 1.5 × 10^6^ cells per well—day 0) in culture media supplemented with 30 ng/mL of *M-CSF*. On day 4, the contents of wells were aspirated and treated with culture media supplemented with the following: 30 ng/mL M-CSF, 30 ng/mL RANKL alone (control) or in addition with either GaAcAc solution, MH, or GaMH (day 4). On day 6, fresh media supplemented with M-CSF and RANKL was added to the differentiating cells. On day 7, the plates were fixed and evaluated for TRAP activity and counted for the number of TRAP-stained multinucleated osteoclasts formed similarly as mentioned above [46].

### Mechanistic assessment of OC differentiation

To investigate the effects on OC differentiation, pre-OC (RAW 264.7) cells were seeded, treated, and differentiated with various treatments as described under OC differentiation. Afterward, cells were lysed with 100 μL of RIPA buffer containing protease inhibitor. The lysates were collected and centrifuged at 1500 RPM for 10 min and analyzed for their protein content via the BCA protein assay kit. Protein samples (20 μg) were separated via 10% SDS-page gel and transferred onto a polyvinylidene fluoride (PVDF) membrane. For an hour, the membrane was blocked with a respective blocking buffer, either 5% BSA or non-fat dry milk. After blocking, the membranes were washed with 1 × TBST buffer and later probed with primary antibodies; NFAT2, TRAP, TRAF6, and cFos with GAPDH as a loading control, diluted with 2% respective blocking buffer overnight at 4 °C, followed by a 1-h incubation with secondary HRP conjugated anti-rabbit IgG secondary antibody. The blots were visualized using the Fluorchem system [47].

### Pit resorption assay for OC function

To assess the effects of various treatments on OC function, we assessed the possible anti-resorptive effect via pit resorption assay [48]. Briefly, bovine cortical bone slices were cleaned and sterilized as per the manufacturer’s instructions and placed in a 24-well plate. The study was carried out using OC derived from either RAW 264.7 differentiation or murine hematopoietic stem cells (mHSCs).

Using OCs from RAW 264.7 cells, the detached pre-OC cells were seeded on top of the sanitized bovine cortical bone slice using 1.5 × 10^5^ cells/cm^2^ supplemented with a complete growth medium on day 0. On the next day (day 1), the media was aspirated from cells followed by treatment with fresh media supplemented with RANKL alone or RANKL with one of the treatments: GaAcAc, MH, or GaMH. Subsequent treatment with complete media containing RANKL occurred every 2 days for 7 days. After 7 days, the wells were aspirated and processed for further analysis.

Separate studies with mHSCs involved seeding mHSCs on top of bone slices at the required amount based on the plate used (96-well plate: 2 × 10^5^ cells/well; 24-well plate: 1.5 × 10^6^ cells/well) in complete AMEM media supplemented with 30 ng/mL M-CSF (day 0). After 3 days, the wells were treated with media supplemented with RANKL and M-CSF with or without GaAcAc alone, MH, or GaMH on day 4. The wells were aspirated on day 6 and replenished with media supplemented with RANKL and M-CSF. The next day, the wells were aspirated and replenished with media containing RANKL; this was repeated for the next 2 days. On day 10, the wells were aspirated and the bone chips were fixed and stained, as reported previously [49].

To assess the osteoclastic resorptive effect of the wells with or without treatment, the bone slices were fixed with 10% formaldehyde and initially TRAP-stained, as reported previously. The bone slices were thoroughly cleaned with cotton swab and strained with 0.5% toluidine blue stain and incubated for 3 min at room temperature. The slices were then washed with water five times and patted dry [10]. The pits formed on the bone slices, depicting OC function, were evaluated.

### Mechanistic assessment of OC function

Cathepsin K (CTSK) assay was conducted during bone resorption studies (as described above) as a useful tool to assess the effects of GaMH treatment on OC function. Supernatant from treated/untreated wells after the third treatment of differentiation factors was collected and spun down at 2000 g for 10 min at 4 °C. The supernatant was then assessed for its CTSK concentration using the mouse CTSK/Cathepsin K Sandwich ELISA kit as per manufacture protocol.

### In vivo retention GaMH

In vivo retention studies were carried out in healthy BALB/c mice using indocyanine green (ICG) as the marker with monitoring via the in vivo imaging system (IVIS) Lumina XRMS series III (PerkinElmer, USA). All animal studies were performed according to a protocol approved by the Institutional Animal Care and Use Committee of Northeast Ohio Medical University. Animals were briefly anesthetized with 2% inhalational isoflurane and administered with 100 µL of ICG solution (20 μg/mL in PBS) as a solution or loaded in GaAcAc-loaded methylcellulose hydrogel (ICG-GaMH).

All treatments were administered by a single periosseous (intramuscular, bone adjacent) injection in the lower right hindlimb. The animals were observed via IVIS at a selected time point to obtain total radiant efficiency of the injection site with an excitation set at 780 nm and emission at 845 nm based on the earlier reported method [50].

### In vivo GaAcAc biodistribution analysis

BALB/c mice were randomly distributed into groups that received 100 µL volume of either GaAcAc solution (200 μg/mL in PBS) or GaMH (loaded with 200 μg/mL GaAcAc). All treatments were administered by a single, periosseous (intramuscular, bone adjacent) injection in the lower right hindlimb. At different time intervals post-injection (2 h and 24 h), mice were sacrificed. From each mouse, we harvested the injection site (lower right hindlimb) as well as a non-injection site (lower left hindlimb). The concentrations of Ga from injection and non-injection sites were analyzed by inductively coupled plasma atomic emission spectroscopy (ICP) (Thermo Scientific iCAP 7400) and normalized by weight of tissue sample. The process of acid digestion of samples was developed using our earlier reported method [51, 52].

### Data analysis

Statistical analysis was carried out using Student’s *t*-test or a single-factor ANOVA. Student–Newman–Keuls post hoc test was applied for multiple comparisons while assessing significance at a *p*-value of < 0.05. The graphs were prepared with GraphPad Prism 9 (GraphPad Software, CA).

## Results

### In vitro characterization of GaAcAc in pre-osteoclastic and pre-osteoblastic cells

**Fig. 1:**
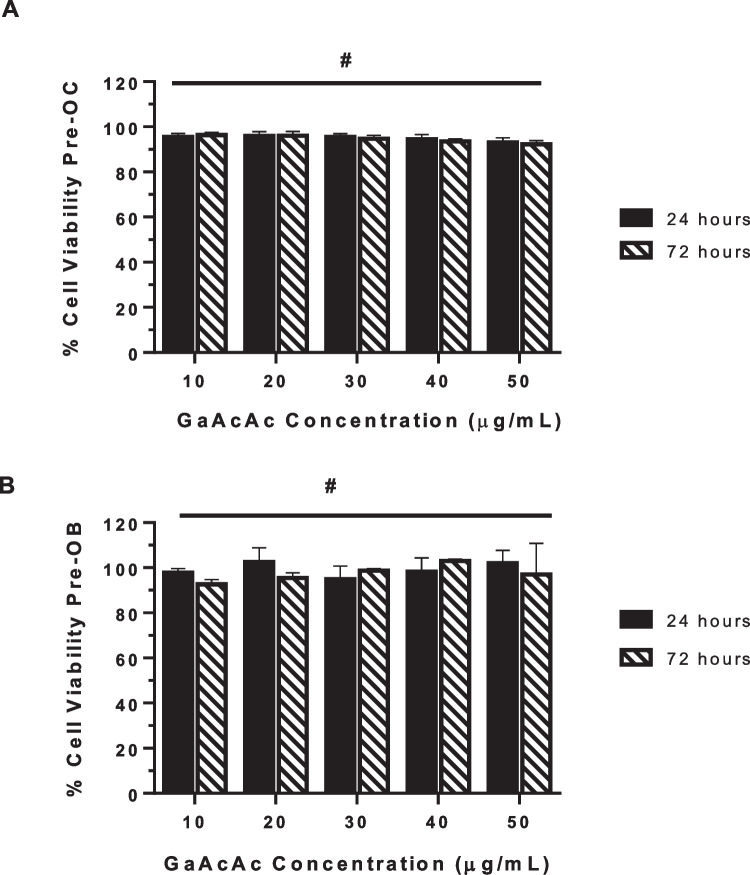
In vitro biocompatibility of GaAcAc. Various concentrations of GaAcAc were incubated (37 °C) with **A** pre-OC cells (RAW 264.7) or **B** pre-OB cells (MC3T3-E1). Percent cell viability was assessed using untreated control cells as a reference. Each data point represents mean ± SD (*n* = 6); #*p>* 0.05

**Fig. 2:**
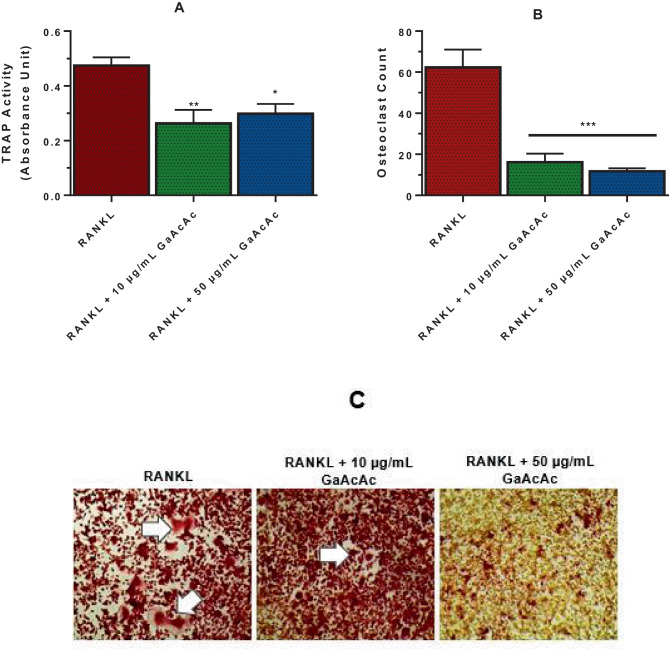
Effects of GaAcAc on OC differentiation. Pre-osteoclastic (RAW 264.7) cells were cultured and differentiated into OC with growth media containing RANKL (30 ng/mL) (positive control). Cells were treated with GaAcAc solution at 10 μg/mL and 50 μg/mL together with RANKL (30 ng/mL). The extent of OC differentiation was assessed by **A** TRAP activity, **B** the number of multinucleated cells (OC) with TRAP-stained containing 3 or more nuclei, and **C** the extent of TRAP staining (white arrows indicating multinucleated cells). Each data point represents mean ± SD; *n* = 6. *p < 0.05, **p < 0.001, and ***p < 0.0001 vs positive control (RANKL)

We investigated the effects of various concentrations of GaAcAc on the viability of pre-osteoclastic (RAW 264.7) or pre-osteoblastic (MC3T3) cells (Fig. 1). GaAcAc concentrations ranging from 10 to 50 µg/mL (37 °C) did not affect the viability of pre-OC (Fig. 1A) or pre-OB (Fig. 1B) cells. Using this GaAcAc concentration window, we explored the effect on OC differentiation as monitored by a notable marker of OC differentiation, tartrate-resistant acid phosphate (TRAP). The differentiation of RAW 264.7 cells into OCs occurred by treatment with RANKL treatment which was supplemented until the differentiation was complete. The differentiated cells were assessed for TRAP activity spectrophotometrically. The trend was substantiated by the number of mature OCs (OC count). The OC count was based on the identification and number of TRAP-stained multinuclear cells (pink color and nuclei ≥ 3) between treatment groups, in comparison to positive control (Fig. 2). GaAcAc treatments were introduced (once) during differentiation with RANKL supplementation. Our data indicate that GaAcAc treatment (at 10 µg/mL and 50 µg/mL) exhibited a remarkable decrease in TRAP activity compared to positive control cells that received RANKL supplementation alone (Fig. 2A; *p* > 0.001).

Further assessment of the extent of OC differentiation was based on the number of TRAP-stained OCs (Fig. 2B). Treatment with 10 µg/mL or 50 µg/mL GaAcAc solution resulted in a significant reduction in the number of OCs after differentiation (*p* < 0.0001, Fig. 2B) when compared to positive control cells that were differentiated without GaAcAc treatment. This inhibitory effect of GaAcAc on OC differentiation was observed in the representative images after differentiation (Fig. 2C) which showed the presence of TRAP-stained multinuclear cells in positive control wells (RANKL) and a decrease in TRAP-stained cells with 10 µg/mL GaAcAc treatment (Fig. 2C). We observed a complete absence of multinucleated and TRAP-stained cells in wells that received the higher GaAcAc concentration (50 µg/mL, Fig. 2C). The data obtained from OC differentiation with mHSCs followed the same trend indicating that GaAcAc treatment suppressed OC differentiation and OC counts (Supplemental Fig. S1). The effect of GaAcAc on OC differentiation using mHSCs was pronounced at 50 µg/mL GaAcAc than at 10 µg/mL (Supplemental Fig. S1). Additional results showed that suppression of OC differentiation by GaAcAc via TRAP activity and OC count was dose-dependent, with a significant inhibition (*p* < 0.0001) observed at GaAcAc concentrations that were greater than 25 μg/mL (Supplemental Fig. S2).

### Preparation and characterization of methylcellulose-based hydrogel

**Fig. 3:**
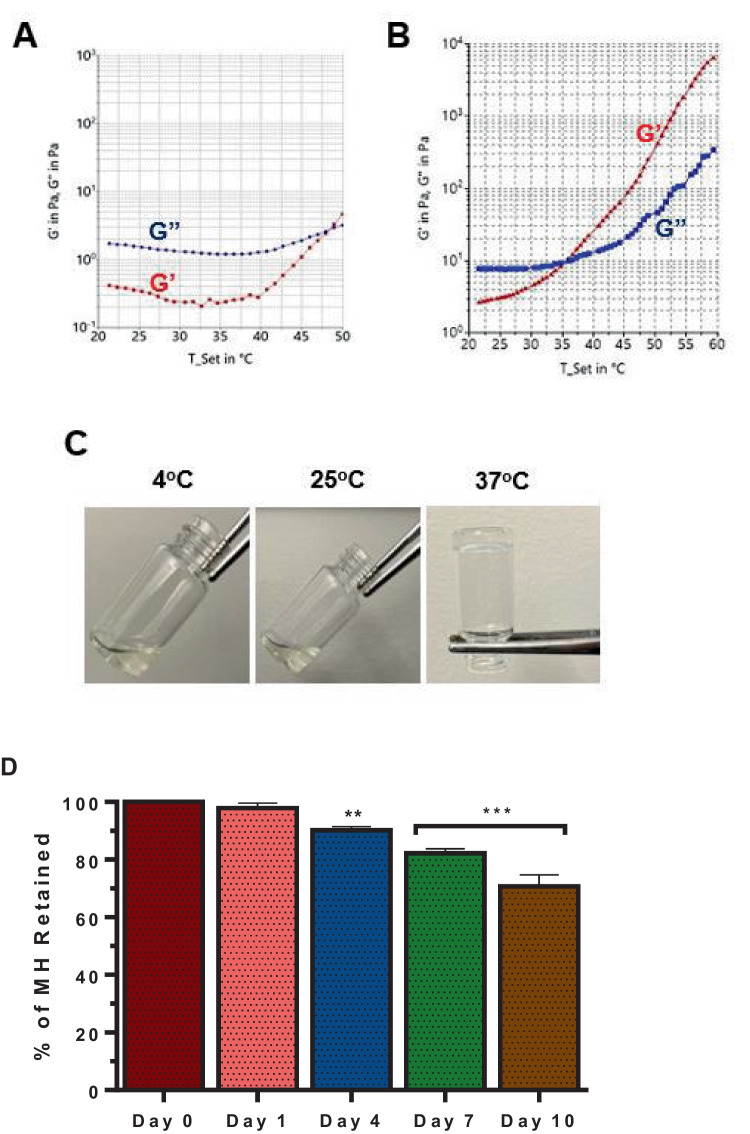
Development and characterization of methylcellulose hydrogels (MH). **A** and **B** Rheological characterization of MH prepared at 6 and 8% w/v of methylcellulose based on storage modulus (G′ as the red line) and loss of modulus (G″ as the blue line) vs temperature (°C). **C** Representative photographs of thermogelling behavior of MH (8% w/v methylcellulose) at 4 °C, 25 °C, and 37 °C. **D** In vitro degradation of hydrogel layer as measured by percentage retention of MH layer when stored with PBS on top of the gel. Each data point represents mean ± SD; *n* = 3 separate formulations. **p < 0.001 and ***p < 0.0001

Different concentrations of methylcellulose (MC), ranging from 2 to 12% w/v, were used to prepare hydrogels. At MC concentrations between 2 and 8% w/v, we prepared suitable hydrogels. MC concentrations above 8% w/v did not result in acceptable hydrogel formulations due to high viscosity that would not be useful practically due to the injectability consideration. All the hydrogels were characterized by thermoreversibility using rheological assessment of storage (G′) and loss (G″) moduli parameters acquired as temperature increased from 25 to 60 °C while noting the temperature at which the moduli will crossover (Fig. 3). At MC concentrations lower than 6% w/v, there was no moduli crossover as temperature increased (Supplemental Fig S3). Hydrogels prepared at MC concentrations 6% w/v exhibited moduli crossover (the gelation temperature) at temperatures around 50 °C (Fig. 3A). Meanwhile, the gelation temperature for hydrogels prepared at MC concentration of 8% w/v was 37 °C.

Further characterization of the thermoreversibility of the lead hydrogel formulation (MH prepared with 8% w/v MC) was carried out using the vial inversion method. The hydrogel was incubated at three different temperatures: 4 °C, 25 °C, and 37 °C. At the same time, we assessed the fluidity by tilting the vial and taking pictures (Fig. 3C). The transition from the solution phase to a solid phase (gel) occurred as the temperature increased as captured for blank hydrogel (MH) (Fig. 3C). In vitro degradation of blank hydrogel (MH) was evaluated with a PBS top layer (37 °C) followed by subsequent measurement of the extent to which the MH hydrogel layer will diminish over the course of 10 days. We observed that the weight of the hydrogel layer decreased consistently over time. The weight on day 0 was set as a reference (100%, Fig. 3D) remained intact (*p* > 0.05) till the following day (day 1). The average percent hydrogel layer retained on day 4 was 90.2% (Fig. 3D) which gradually decreased to an average of 70% on day 10, indicating a loss of 15% hydrogel weight by day 10 (Fig. 3D). The biocompatibility of the hydrogel (MH) was demonstrated based on the viability of pre-OCs (RAW 264.7) and pre-OB (MC3T3) cells using MTT or neutral red dye uptake methods (Supplemental Fig. S4A-D).

### Preparation and characterization of GaAcAc-loaded hydrogels

**Fig. 4:**
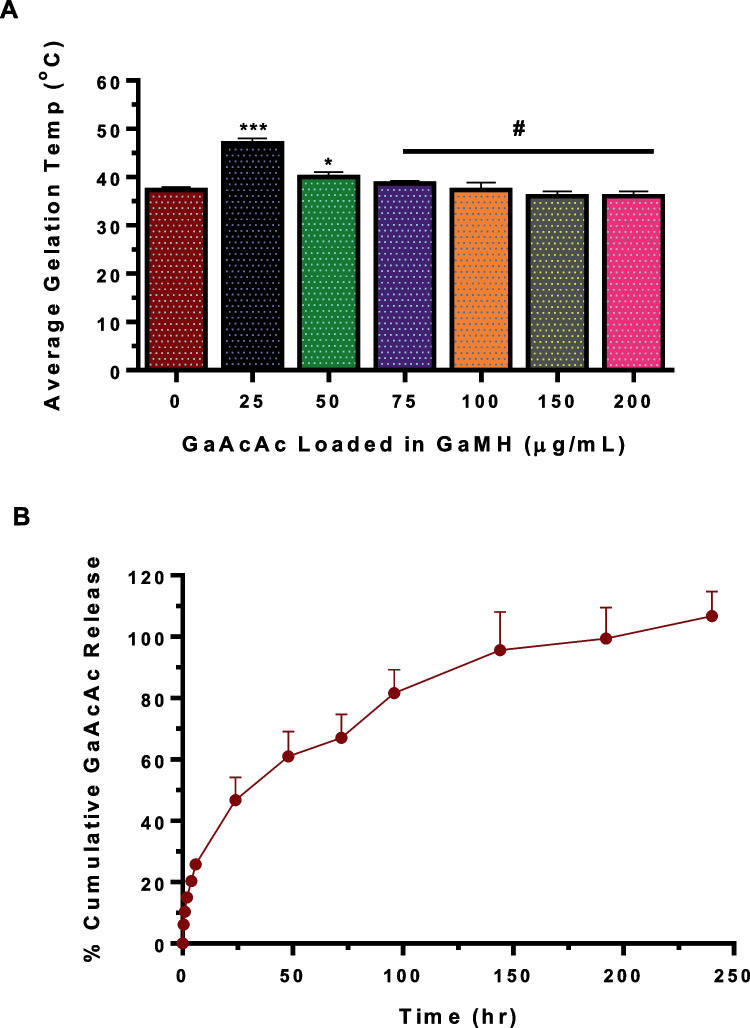
Characterization of GaMH (GaAcAc-loaded methylcellulose hydrogels). **A** Effects of various concentrations of GaAcAc on gelation temperatures of GaMH as obtained from rheological measurements. **B** In vitro release profile of GaAcAc from GaMH. Each data point represents mean ± SD; *n* = 3–4, #*p* > 0.05, *p < 0.05, and ***p < 0.0001 vs positive control (blank hydrogels)

Hydrogels loaded with different concentrations of GaAcAc (GaMH), ranging from 25 to 200 µg/mL, were prepared and characterized. Rheological parameters, storage (G′), and loss (G″) moduli, with increased temperature (20 to 60 °C), were obtained. The moduli readings are shown in the supplemental file (Supplemental Fig. S5A-E). From the moduli readings, we obtained the average gelation temperature of GaMH at different concentrations of GaAcAc. At GaAcAc concentrations (25–50 µg/mL), the average gelation temperatures increased slightly, compared to blank hydrogels (*p* < 0.05) and later remained comparable to blank hydrogels. As we increased GaAcAc concentrations from 75 to 200 µg/mL, the average gelation temperatures were comparable to blank hydrogels (*p* > 0.05; Fig. 4A). GaMH formulation loaded with a GaAcAc concentration of 100 μg/mL was selected as the lead for subsequent studies. Further characterization of the thermoreversibility of the selected GaMH (containing GaAcAc 100 µg/mL) was carried out using the vial inversion method with incubation at three different temperatures. The images showed fluidity of GaMH at 4 °C and 25 °C, consistent with the solution phase, but not at 37 °C, consistent with the gel or solid phase (Supplemental Fig. S5F). The rate of GaAcAc release from GaMH was evaluated by maintaining the hydrogel formulations at the gel phase (37 °C) throughout the study with gentle shaking at 50 RPM with a PBS layer on top. Within 6 h, we observed an average of 25% GaAcAc release which gradually increased to an average of 46% GaAcAc release by 24 h. The rate of GaAcAc release slowed down afterward resulting in an average 61, 67, and 81% at 48, 72 and 96 h, respectively (Fig. 4B).

### Efficacy assessment in OC differentiation and function

**Fig. 5:**
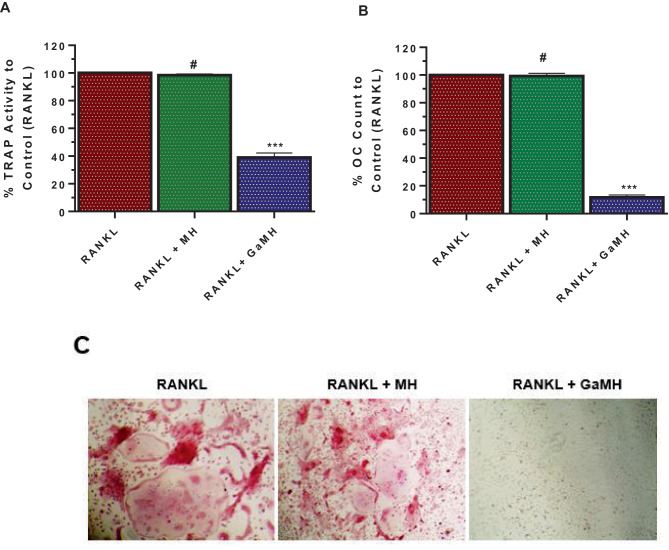
Effects of GaMH on OC differentiation. Murine hematopoietic stem cells were collected and differentiated to OCs with different treatments of RANKL and M-CSF alone (positive control) or together with MH or GaMH (containing GaAcAc 10 µg/mL). At the end of differentiation, cells were assessed for **A** TRAP activity and **B** number of multinucleated cells (OC) with TRAP-stained containing with 3 or more nuclei. Only cells with 3 or more nuclei and TRAP stained, i.e., pink color, were counted, and **C** extent of TRAP staining. Each data point represents mean ± SD; *n* = 3, #*p*> 0.05 and ***p < 0.0001versus positive control (RANKL)

The initial observation showed that blank hydrogels (MH) when added during differentiation of mHSCs to OCs did not interfere with the differentiation process (Fig. 5). TRAP activity and OC counts following M-CSF and RANKL supplementation with MH treatment were comparable to the observation with the positive control cells that were differentiated without MH treatment (Fig. 5A and B). A similar trend was observed with differentiation of RAW 264.7 cells to OCs with or without MH treatment (Supplemental Fig. S6). However, with GaMH treatment, there was a significant decrease in the TRAP activity and OC counts compared to positive control cells (*p* < 0.0001, Fig. 5A and B). Further, the presence of TRAP-stained multinuclear cells was unaffected in wells treated with MH; however, the wells treated with GaMH significantly decreased the presence of any multinucleated cells (Fig. 5C and Supplemental Fig. S6).

**Fig. 6:**
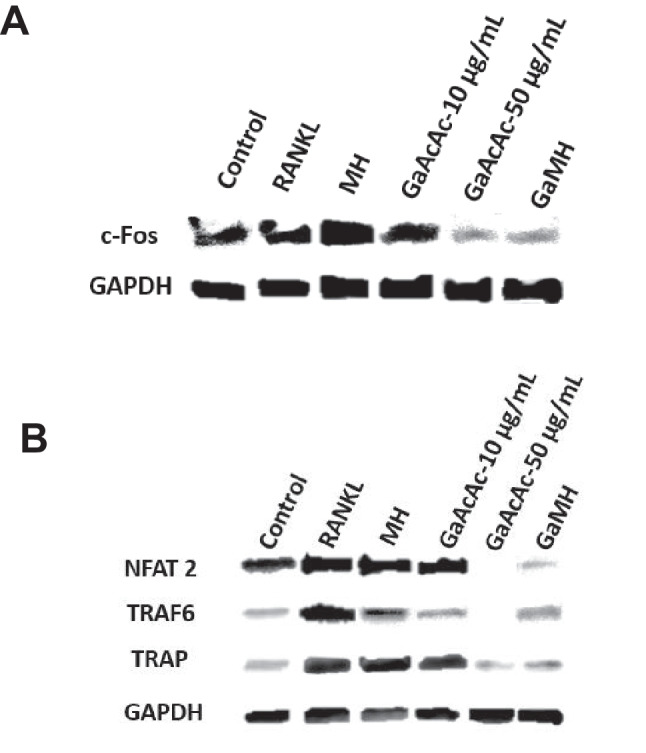
Mechanistic assessment of OC differentiation markers. Expression of key markers of OC differentiation as analyzed by western blotting **A** *cFos* as well as **B** NFAT2, TRAF6, and TRAP. RAW 264.7 cells were differentiated into OCs with treatment with RANKL alone (control) or RANKL together with any of the following: GaAcAc solution (10 μg/mL and 50 μg/mL), blank hydrogel (MH), and GaMH (loaded with 10 µg/mL)

To analyze the mechanistic basis of the observed trends in OC differentiation and function, we performed western blotting analyses focusing on key markers involved in OC differentiation such as TRAP, NFAT2, and cFos (Fig. 6). We observed that treatment with blank hydrogel (MH) during differentiation did not affect the expression of any of these markers compared to the positive control cells (with RANKL supplementation alone). Treatment with 10 µg/mL GaAcAc solution during differentiation resulted in a slight decrease in expression levels of the markers when compared to positive control. Meanwhile, treatment with 50 µg/mL GaAcAc or GaMH (loaded with 10 µg/mL GaAcAc) resulted in downregulation of expression levels of these markers when compared to a positive control (Fig. 6A and B).

**Fig. 7:**
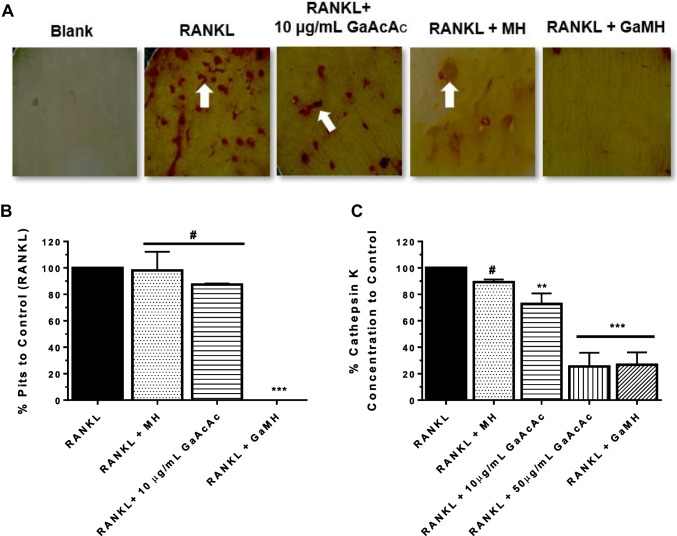
Ex vivo characterization of osteoclastic bone resorption after GaMH treatment. RAW 264.7 cells were differentiated to OCs on bovine cortical slices with RANKL treatment alone (positive control) or RANKL treatment together with any of the following: GaAcAc solution (10 μg/mL) or blank hydrogel (MH) or GaMH (containing 10 µg/mL GaAcAc). After OC differentiation process, the bone slices were stained via **A** TRAP for the presence of differentiated OCs (white arrows) and **B** stained with 0.5% toluidine blue to count for osteoclastic bone resorbed pits on bone slices after various treatments. **C** CTSK analysis by ELISA of cell media during OC differentiation on bone slices. Each data point represents mean ± SD; *n* = 3. **p < 0.001, ***p < 0.0001 vs positive control (RANKL)

Further assessments using bone resorption assays were performed by differentiating pre-OC cells (RAW 264.7) into OCs on a bone matrix (bovine cortical bone slices) in an ex vivo setting. After OC differentiation, the bone slices were initially stained with TRAP and later with 0.5% toluidine blue stain to assess the pits formed by activated OC upon bone matrix degradation (Fig. 7A). With the treatment with 10 µg/mL GaAcAc solution, the resorption pits visible in positive control cells were slightly reduced (Fig. 7A; *p* > 0.05). Treatment with GaMH (containing 10 µg/mL) during OC differentiation resulted in lack of visible resorption pits on bone slices (Fig. 7B; *p* < 0.0001) which agrees with the observation on bone resorption assay using OCs from mHSCs (Supplemental Fig. S7).

During the pit resorption assay, cell media was aliquoted after OC activation and assessed for CTSK marker using ELISA. MH treatment did not affect levels of CTSK after OC activation from RAW 264.7 cells while treatment with GaAcAc solution (10 µg/mL) caused a slight reduction (1.4 times reduction) in CTSK concentration (Fig. 7C). The level of CTSK was markedly reduced (4 times reduction) upon treatment with 50 µg/mL GaAcAc solution or GaMH (containing GaAcAc at 10 µg/mL) (Fig. 7C; *p* > 0.001). From additional studies on differentiation of mHSCs to OCs on bone slices, we observed a pronounced reduction in CTSK levels with GaMH treatment compared to GaAcAc solution (Supplemental Fig. S7C).

### In vivo characterization of GaMH

**Fig. 8:**
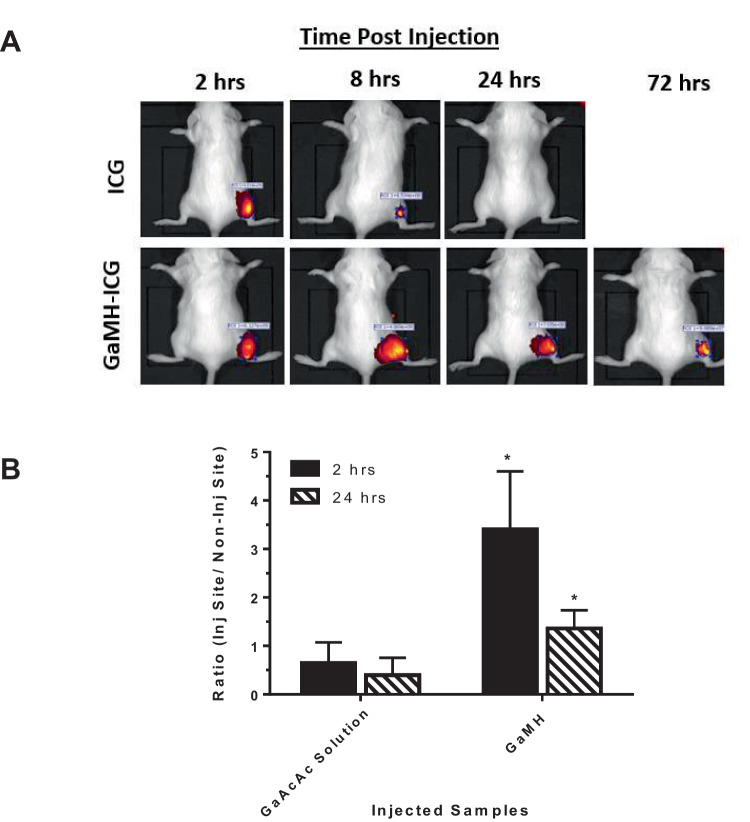
In vivo bio-retention of GaMH after injection in BALB/c mice. **A** Representative IVIS images of BALB/c mice after injection of fluorescent-labeled GaMH (GaMH-ICG) or ICG solutions into the lower right hindlimb (periosseous) of BALB/c mice. Animals in all groups were imaged at different time intervals after injection. **B** Concentration of gallium (Ga) retained at injection site in BALB/c mice. Mice were injected with GaAcAc solution or GaMH as a single periosseous injection into the lower right hindlimb of BALB/c mice. The extent of Ga retention in each mouse (2 h and 24 h post-injection) was expressed as a ratio of concentration of Ga at the injection site (Inj. Site) versus a non-injection site (Non-Inj Site) when measured by ICP. Each data point represents mean ± SD; *n* = 3 mice (*p < 0.05)

For the bio-retention studies, GaMH was fluorescently labeled with ICG (ICG-GaMH) and administered by a single periosseous injection to the lower right hindlimb of BALB/c mice. Similarly, aqueous solutions of PBS (control) or ICG were administered. All mice were imaged via IVIS at various time points (Fig. 8). The fluorescent intensity from mice administered with aqueous solutions of ICG diminished by 8 h post-injection and completely disappeared by the time we acquired the images at 24 h post-injection. In comparison, the fluorescence, from ICG-GaMH, was retained throughout the image acquisition at 72 h (Fig. 8). We conducted a follow-up study involving injecting (a single periosseous injection) an equal dose of GaAcAc either as GaAcAc solution or GaMH. Mice were terminated at 2 h and 24 h post-injection. The concentration of Ga in each tissue sample was measured by ICP. The ratio of concentration of Ga at the injection site versus a non-injection site was obtained. At 2 h post-injection, the ratio of Ga retained at the injection site with GaMH was 5 times higher than when GaAcAc solution was administered. At 24 h post-injection, the ratio of Ga retention was 3.4 times higher with GaMH than with GaAcAc solution (Fig. 8B).

## Discussion

Bone resorptive disorders have posed a major health risk in most of the population, especially geriatrics. The detrimental effects of these disorders originate from weakened skeletal structures leading to fractures and delayed fracture healing [53]. The pathological basis can be attributed to an imbalance between OC and OB regulation, specifically, an enhanced OC differentiation and function that will ultimately favor bone resorption [1]. Hence, strategies that target OC differentiation and resorption (such as anti-resorptive treatment) without affecting OB functioning will be beneficial in treating bone resorptive disorders. Additionally, treatment regimens that mitigate off-target effects are more favorable and may have clinical utility [54]. Thus, the need to explore alternative therapeutic approaches that maximize safety and efficacy remains at the forefront of drug development.

Earlier studies have shown that gallium compounds (gallium nitrate) can effectively inhibit OC differentiation and function without affecting OBs [27, 55]. In various gallium compounds, we are of the opinion that the ligands associated with the parent gallium ion will play an influential role in dictating the resultant physico-chemical properties such as water solubility. In this work, we investigated the effect of GaAcAc against OC differentiation and osteoclastic bone resorption. GaAcAc, a poorly water soluble compound, has gallium ion complexed with three molecules of acetylacetonate ligand group [52]. We believe that suitable delivery systems will be necessary to realize the therapeutic potentials of gallium compounds by making it possible to deliver effective doses and overcome challenges associated with poor retention at the desired site and off-target distribution. Initial studies showed that GaAcAc is biocompatible with bone cells (pre-OCs and pre-OBs) at 10–50 µg/mL concentration range (Fig. 1). Further, we demonstrated the inhibitory effects of GaAcAc on OC differentiation processes (Fig. 2 and Supplemental Figs. S1 and S2), exhibiting a concentration-dependent effect on TRAP activity and OC counts which agrees with earlier studies with gallium nitrate [27].

Intending to design and develop a suitable delivery system for GaAcAc, we broadly screened a few delivery platforms and assessed the extent to which these will interfere with OC differentiation process (data not shown) before we eventually selected cellulose-derived thermosensitive gel (methylcellulose-based hydrogel, MH). MH comprises a well-knit mesh-like framework formed by the methoxyl groups [40], which we expect can entrap a poorly water soluble drug such as GaAcAc with subsequent release in a controlled manner. The principle behind the solution-to-gel (sol–gel) phase transition of this unique cellulose-based hydrogel is due to the temperature-dependent fluctuating water molecules encasing the hydrophobic methoxyl groups. As the temperature dips below physiological temperature, the water molecules remain intact, leaving the gel in a solution state. In contrast, increasing temperature releases the water molecules, favoring the solid-state gel [40, 41].

We fabricated and characterized MH using the dispersion method at various concentrations of MC. The thermoresponsive property of the hydrogel as a drug delivery system facilitates ease of injection by remaining in solution phase at 25 °C, while conversion to solid at the injection site will favor bio-retention and less off-target distribution. We applied rheological evaluation that explores changes in storage (G′) and loss (G″) moduli with increase in temperature. The storage modulus (G′) measures the elasticity of the material and its ability to store energy, whereas the loss modulus (G″) is a measure of the ability to release energy. By measuring the G′ and G″ moduli over a temperature range, we could assess the structural state of the hydrogel at a different temperature, the sol–gel transition of the hydrogel, and the gelation temperature. The liquid behavior is associated with a G″ (loss of modulus) that is higher than G′ (storage modulus). At the gelation temperature and beyond, the storage modulus (G′) is greater than the loss modulus (G″), which reflects a change to a gel phase. The pattern correlates with previous studies in the rheological assessment of the thermoreversible hydrogels.

The rheological evaluation of hydrogels showed that at MC concentrations below 6% w/v, there was no crossover between G′ and G″, indicating lack of thermoresponsive behavior. The moduli crossover, signaling gelation occurred in hydrogels prepared with MC concentration of 6%; however, gelation temperature was close to 50 °C (Fig. 3A). Therefore, hydrogels prepared at 8% w/v MC was selected for GaAcAc delivery because the gelation temperature of 37 °C is ideal for an injectable application. We could not obtain viable hydrogels at MC concentrations above 8% partly due to high viscosity of the MC solution. Further assessment showed that hydrogels prepared with MC are biocompatible and undergo a gradual loss of the hydrogel layer (Fig. 3D; Supplemental Fig. S4). These characteristics agree with earlier reports that the internal framework of MC-based hydrogels can degrade over time [56].

Subsequent studies showed that a broad concentration range of GaAcAc (25–200 µg/mL) can be loaded onto the hydrogels while maintaining the thermoresponsive properties (Fig. 4A). The release kinetics of GaMH showed that GaAcAc was released in a controlled manner over time, comprised of an initial fast release phase that was followed by a slow-release phase (Fig. 4B). The trend suggests that GaAcAc encapsulated in the hydrogel was initially released due to its presence on the surface (burst release), followed by the gradual release that could be linked to continued degradation of the gel. Further elucidations are required to understand the complete mechanism behind GaAcAc release and its stability; however, our data shows that GaAcAc can be effectively loaded into a methylcellulose-based hydrogel (MH), and we are unaware of any other studies that have focused on delivery systems of gallium compounds for application in bone disorders.

The next parameter assessed the OC inhibitory effects of hydrogels loaded with GaAcAc (GaMH) compared to GaAcAc solution (not loaded in hydrogels). In our initial investigation, both GaAcAc alone and GaMH significantly inhibited OC differentiation (Figs. 2 and 5, Supplemental Figs. S1 & S6). We verified that blank hydrogels (MH) did not interfere with OC differentiation, indicating that the efficacy of GaMH could be attributed to GaAcAc loading. Overall, our findings suggest that hydrogels loaded with GaAcAc (GaMH) showed better efficacy in hindering OC differentiation when compared to GaAcAc solution. To further characterize the effect of GaAcAc and GaMH, we performed western blotting analyses of key markers involved in OC differentiation and function in bone resorption. Previous studies have reported that complementary binding of RANKL to RANK receptor triggered a myriad signaling cascade involving the following markers: cFos, TRAF6, TRAP, and NFAT2 [27, 57]. Increasing the concentration of GaAcAc from 10 to 50 µg/mL resulted in marked downregulation of all the expression of all the identified OC differentiation markers (Fig. 6). It is noteworthy that GaMH containing 10 µg/mL was superior to GaAcAc solution at the same concentration when considering the expression levels of OC differentiation markers (Fig. 6).

To further assess the effect of GaAcAc solution and GaMH on OC function, we performed resorption studies (ex vivo) using bovine bone slices (Fig. 7 and Supplemental Fig. S7). In the study, we observed the extent to which differentiated OCs will resorb bone slices. Upon staining, the pits on the bone slices were visually clearer and facilitated a quantitative analysis of treatment with GaAcAc versus GaMH. Wells treated with MH were unaffected; however, there was a slight decrease in the pits formed in wells treated with 10 µg/mL GaAcAc. Treatments with GaAcAc (50 µg/mL) and GaMH significantly decreased the number of pits formed due to the inhibition of the activated OCs compared to the positive control (Fig. 7B and Supplemental Fig. S7B). This OC inhibitory trend of GaMH in vitro and ex vivo coincides with the trend observed initially on GaAcAc (50 µg/mL) treatment against differentiated OCs (Fig. 7A and Supplemental Fig. S7A) [24, 58, 59]. The concentration of CTSK release during the resorptive process was analyzed to confirm the anti-resorptive trend further. Bone resorption involves release of CTSK by activated OCs that is subsequently secreted to degrade the organic matrix [10, 60]. Normally, the release of CTSK is directly proportional to OC resorption as a viable marker of the extent of OC function [61]. In this case, a decrease in the levels of secreted CTSK will correspond to inhibition of bone resorption. Reduction in levels of CTSK was observed in treatments with GaAcAc solution (10 µg/mL and 50 µg/mL) in a dose-dependent manner. Our observation showed that GaMH (containing GaAcAc 10 µg/mL) performed better than GaAcAc solution at the same concentration level (Fig. 7C and Supplemental Fig. S7C) in reducing CTSK concentration, which corresponds with inhibiting OC function. The performance of GaMH compared to GaAcAc solution could be attributed to the ability to control the release of GaAcAc and maximize its availability and efficacy. We believe that the thermoresponsive behavior of MH will be closely linked to the bio-retention property and the ability to achieve localized therapeutic effect, and maximize the effect at the desired site while limiting off-target effects [62, 63]. Our initial assessment demonstrated the bio-retention of GaMH following periosseous injection in BALB/c mice as measured by close monitoring of injection site for fluorescence and concentration of gallium via ICP.

In conclusion, we assessed the potential of a new gallium compound (GaAcAc) as an anti-resorptive therapeutic agent. The stability and thermoresponsive behavior of MC-based hydrogel delivery systems were demonstrated. MC-based hydrogels showed attractive qualities in GaAcAc delivery such as (a) GaAcAc loading onto the hydrogels did not interfere with the thermoreponsive behavior, (b) the release of GaAcAc occurred in a controlled manner to maximize exposure with OCs, (c) based on expression of key markers, GaAcAc released from hydrogels was effective in inhibiting OC differentiation and function, (d) hydrogels loaded with GaAcAc (GaMH) showed superior performance in inhibiting OC differentiation and function compared to GaAcAc solution, and (e) GaMH demonstrated ability to achieve longer bio-retention at the site of injection compared to GaAcAc solution, which could maximize exposure at the site of action while minimizing off-target distribution.

## Supplementary Information

Below is the link to the electronic supplementary material.

Supplementary file1 (DOCX 2474 KB)

## Data Availability

The datasets generated during and/or analyzed during the current study are available from the corresponding author on reasonable request.
