# Supplementary material for: Efficacy assessment of methylcellulose-based thermoresponsive hydrogels loaded with gallium acetylacetonate in osteoclastic bone resorption
Source: Drug Deliv Transl Res. 2023 Apr 4;13(10):2533–49. doi: 10.1007/s13346-023-01336-5 (PMC10469133; doi:10.1007/s13346-023-01336-5)
Supplement: Supplementary file 1 — Supplementary file1 (DOCX 2474 KB) [file 13346_2023_1336_MOESM1_ESM.docx]

Supplemental File (Online Resource)

Submitted to: ***Drug Delivery and Translational Research***

*March 16, 2023*

***Efficacy Assessment of Methylcellulose-Based Thermoresponsive Hydrogels Loaded with Gallium Acetylacetonate in Osteoclastic Bone Resorption***

Pratyusha Ghanta^1,2^, Timothy Winschel^1^, Evin Hessel^1^, Oluyinka Oyewumi^3^,

Tori Czech^1^ and Moses O. Oyewumi^1,2^*

^1^Advanced Drug Delivery Laboratory, Department of Pharmaceutical Sciences,

College of Pharmacy, Northeast Ohio Medical University, Rootstown, OH 44272, USA

^2^Department of Biomedical Sciences, Kent State University, Kent, OH 44240

^3^Department of Geological Sciences, Central Connecticut State University,

New Britain, CT 06050

*Correspondence:

Moses Oyewumi, B. Pharm, Ph.D.

Chair and Associate Professor

Department of Pharmaceutical Sciences

Northeast Ohio Medical University

4209 State Route 44

Rootstown, OH 44272

USA

Tel: 1-330-325-6669

Email: [moyewumi@neomed.edu](mailto:moyewumi@neomed.edu)

**Table S1: GaAcAc Concentration Expressions (µg/mL and µM)**

| **GaAcAc Concentration (Expression as µg/mL)** | **Corresponding GaAcAc Concentration (Expression as µM)** |
| --- | --- |
| 5 | 13.60 |
| 10 | 27.24 |
| 15 | 40.87 |
| 20 | 54.49 |
| 25 | 68.11 |
| 30 | 81.73 |
| 35 | 93.35 |
| 40 | 108.98 |
| 45 | 126.60 |
| 50 | 136.22 |

**Figure S1**


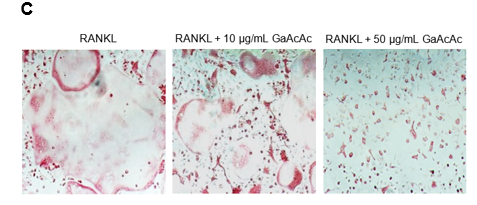


**Figure S1:** **Effects of GaAcAc on OC differentiation using murine hematopoietic stem cells:** Bone marrow cells were flushed and incubated to obtain murine hematopoietic stem cells. Which were then cultured and differentiated into OC with growth media containing RANKL (30 ng/mL) & MCSF (30 ng/mL) (positive control). Cells received GaAcAc treatments at 10 & 50 μg/mL together with RANKL (30 ng/mL) & MCSF (30 ng/mL). The extent of OC differentiation was assessed by **(A)** TRAP activity, **(B)** the number of multi-nucleated cells. Each data point represents mean ± SD; n=4. # p>0.05, ***p<0.0001 vs positive control.

**Figure S2**

**Figure S2: Dose-Dependent effects of GaAcAc on OC Differentiation:** RAW cells were cultured and differentiated into OC with growth media containing RANKL (30 ng/mL) (positive control). Cells received GaAcAc treatments at various concentrations from 5 to 50 μg/mL together with RANKL (30 ng/mL). The extent of OC differentiation was assessed by **(A)** TRAP activity, **(B)** the number of multi-nucleated cells (OC) with TRAP-stained containing 3 or more nuclei. Each data point represents mean ± SD; n=4. *p<0.001, **p< 0.0001 and ***p<0.0001 vs positive control.

**Figure S3**


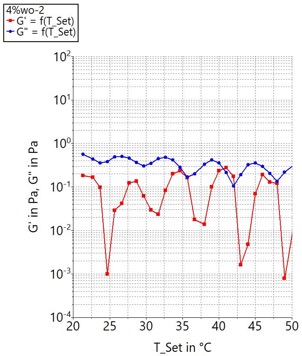


A

B

**Figure S3: Rheological Characterization of Methylcellulose Hydrogels (MH): (A & B)** Rheological assessment of MH prepared at various concentrations: **(A)** 2 % w/v **(B)** 4% w/v methylcellulose hydrogel methylcellulose based on storage modulus (G’) and loss of modulus (G”) over the temperature range 20- 60º C.

**Figure S4**

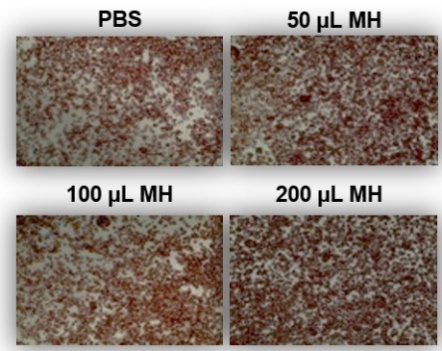


**Figure S4: Figure S4: Biocompatibility Analysis of MH: (A)** MTT analysis of MH leachate against RAW 264.7 cells **(B)** MTT analysis of MH against MC3T3 cells, **(C)** MTT analysis of MH against RAW cells, **(C)** Neutral Red uptake of RAW264.7 cells treated with different volumes of MH. Each data point represents mean ± SD; n=3-4. #p>0.05,***p<0.0001.

**Figure S5**


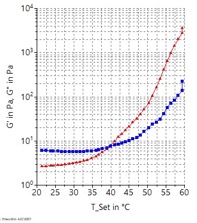

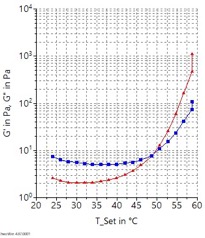

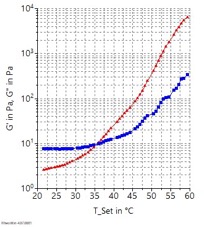

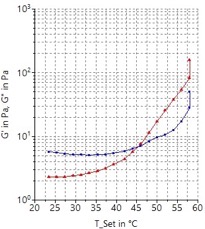


**A**

**B**

**C**

**D**


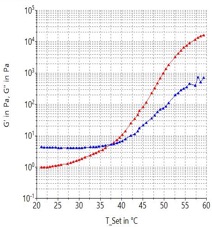


**E**

4º C

25 º C

37 º C


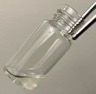

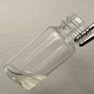

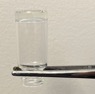


**F**

**G**’

**G**’

**G”**

**G**’

**G”**

**G”**

**G**’

**G**’

**G”**

**G”**

**Figure S5: Rheological analysis of different concentrations of GaAcAc in 8% MH via Gelation temperature:** Cross over of G’ and G” over a range of temperature (20-60 °C), i.e., gelation temperature was observed for: **(A)** 25 µg/mL GaAcAc **(B)** 50 µg/mL GaAcAc **(C)** 75 µg/mL GaAcAc **(D)** 100 µg/mL GaAcAc **(E)** 200 µg/mL GaAcAc

**Figure S6**

**Figure S6: Effects of GaMH in Osteoclastogenesis:** Pre-osteoclastic cells (RAW 264.7 cells) were differentiated to OCs with growth media containing RANKL alone (30 ng/mL), positive control or RANKL with MH as well as RANKL + GaMH (delivering GaAcAc 10µg/mL). At the end of differentiation, the cells were assessed for **(A)** TRAP activity and (B) the number of multi-nucleated cells (OC) with TRAP-stained containing three or more nuclei. Only cells with three or more nuclei and TRAP stained, i.e., pink color, were counted. Each data point represents mean ± SD; n=3. ***p<0.0001, #p˃ 0.05 versus positive control.

**Figure S7**


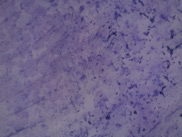

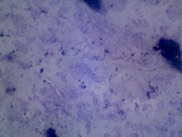

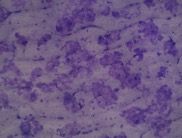


RANKL

RANKL + MH

RANKL +10 µg/mL GaAcAc


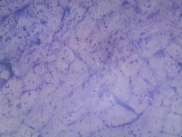


RANKL + GaMH


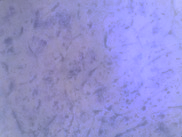


RANKL + 50 µg/mL GaAcAc

**A**

**Figure S7: *Ex-vivo* Characterization of OC Function to Resorb Bone:** Hematopoietic stem cells were collected and differentiated to osteoclasts on bovine cortical slices, and well contents were treated with growth media supplemented with RANKL (30 ng/mL) and MCSF (30 ng/mL) without or with GaAcAc (10 & 50 μg/mL) or MH or GaMH, to assess their effect on osteoclastic pit resorption. From the 6^th^ day onwards to day 10, the well content was aspirated and treated with growth media with only differentiation factors. The slices were stained via **(A)** Pictorial representation of the effect of GaAcAc, MH & GaMH against RAW cell differentiated osteoclasts on bovine cortical bone slices stained with 0.5% toluidine blue to represent the osteoclastic pits formed (white arrows). **(B)** Quantitative assessment of osteoclastic pits formed. **(C)** CTSK analysis of OC differentiating supernatant via ELISA. Each data point represents mean ± SD; n=3. # p>0.05, *p<0.05, **p<0.0001 &*** p<0.0001 vs positive control.

**Figure S8**


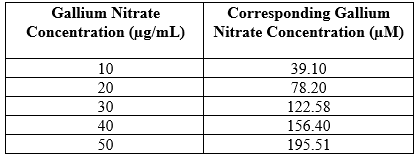


**D**

**Figure S8: Initial screening of different concentrations of gallium nitrate (GaN) on OC differentiation using RAW 264.7 cells.** RAW cells were cultured and differentiated into OC with growth media containing RANKL (30 ng/mL) (positive control). Cells received gallium nitrate treatments at various concentrations from 5 to 50 µg/mL (19 µM- 195 µM) together with RANKL (30 ng/mL). The extent of OC differentiation was assessed by **(A)** TRAP activity, **(B)** the number of multi-nucleated cells (OC) with TRAP-stained containing 3 or more nuclei. Each data point represents mean ± SD; n=4. #p>0.05, *p<0.01, **p< 0.001 and ***p<0.0001 vs positive control.(C) Cell viability after incubating (37^o^C) various concentration of gallium nitrate with pre-OC cells (RAW 264.7). Each data point is a percentage based on untreated control cells that served as a reference. **(D)** A summary table of gallium nitrate concentration expression in terms of µg/mL and µM.
